# Genomic features of liquid biopsies from patients with prostate cancer with and without ductal adenocarcinoma

**DOI:** 10.1002/2056-4538.70026

**Published:** 2025-04-02

**Authors:** Qiyu Zhu, Ting Wang, Yifu Shi, Xiuya Zhou, Dingbang Liu, Junru Chen, Haoyang Liu, Fengnian Zhao, Chenhao Xu, Yuntian Chen, Jiayu Liang, Ni Chen, Pengfei Shen, Hao Zeng, Jinge Zhao

**Affiliations:** ^1^ Department of Urology Institute of Urology, West China Hospital, Sichuan University Chengdu PR China; ^2^ Department of Nursing West China Hospital, Sichuan University Chengdu PR China; ^3^ Department of Pathology West China Hospital, Sichuan University Chengdu PR China; ^4^ Department of Radiology West China Hospital, Sichuan University Chengdu PR China

**Keywords:** ductal adenocarcinoma, FOXA1, AR pathway

## Abstract

Ductal adenocarcinoma of the prostate (DA) is relatively rare and highly co‐existent with prostate adenocarcinoma (AC). This study aimed to investigate the distinctive genomic profiles of patients with DA compared to those without. Blood samples were obtained from 144 patients (36 with DA and 108 without DA) who were diagnosed from 2017 to 2023 at West China Hospital. We performed cell‐free DNA sequencing to investigate the genomic differences between patients with DA [DA(+)] and those without [DA(−)], and explored the potential associations between their mutational status and prognosis. Pathogenic and likely pathogenic alterations were included for analysis. We identified that AR pathway [16/36 (44.4%) versus 24/108 (22.2%), *p* = 0.017] and WNT pathway [6/36 (16.7%) versus 5/108 (4.6%), *p* = 0.029] mutations were significantly enriched in DA(+) compared to DA(−) patients. Mutation of *FOXA1*, as a key component of the AR pathway, demonstrated markedly higher prevalence in the DA(+) over the DA(−) cohort [25% (9/36) versus 4.6% (5/108), *p* = 0.0012]. The DNA damage repair mutation rate and the homologous recombination repair deficiency scores appeared to be comparable between the DA(+) and DA(−) patients. In the metastatic population, DA was characterized by a higher speckle‐type POZ protein (*SPOP*) mutation rate. *TP53* mutation was associated with a deteriorating prognosis for both DA(+) and DA(−) patients in terms of castration‐free survival. In conclusion, our findings provide further genomic insights into prostate cancer with ductal morphology and are instructive for the diagnosis and treatment of DA.

## Introduction

Ductal adenocarcinoma of the prostate (DA), featured by a papillary structure or cribriform glands lined with tall columnar pseudostratified epithelial cells, occurs in approximately 10% of prostate cancer (PCa) patients while coexisting with acinar adenocarcinoma (AC) [1]. DA exhibits a high rate of de novo visceral metastases with a relatively low baseline prostate‐specific antigen (PSA) level, leading to diagnostic challenges and poor patient prognosis [1, 2].

Previous studies showed that, in the M0 cohort, the presence of DA was independently associated with a poor response to conventional hormone therapy [3, 4]. Conversely, among the metastatic population undergoing androgen deprivation therapy, DA appeared not to be correlated with adverse clinical outcomes [5]. A multi‐omics analysis by Gillard *et al* reported comparable AR activity between DA and AC [6], while our earlier work indicated that the presence of DA could serve as a potential prognostic marker for advanced PCa, inducing responsiveness to the potent androgen‐synthesis inhibitor, abiraterone [7]. These findings underscore the importance of elucidating the molecular mechanism of DA pathogenesis, especially the AR pathway. However, contemporary genomic research on DA is scarce, possibly due to its rarity. Several studies have conducted tissue‐based genomic sequencing on DA, while some of the results appeared to be heterogeneous, typically regarding the abundance of DNA damage repair pathway mutations [8, 9].

Liquid biopsy has demonstrated advantages over traditional tissue‐based sequencing in providing dynamic molecular insights into PCa. It effectively captures intratumor heterogeneity, which cannot be accurately assessed by a single biopsy specimen, especially in metastatic cases [10]. Additionally, liquid biopsy is less invasive and offers highly concordant mutational results when compared to traditional tissue‐based genomic sequencing [11].

Therefore, the aim of this study was to compare the genomic profiles of patients with [DA(+)] and without DA [DA(−)] using cell‐free DNA (cfDNA) sequencing. Additionally, we explored potential cfDNA‐derived prognosticators for both DA(+) and DA(−).

## Materials and methods

### Patients and sample collection

In this study, we retrospectively collected 144 PCa samples from 2017 to 2023 at West China Hospital, including 36 patients with DA and 108 without. Written informed consent was obtained from each patient for sample collection, regular follow‐up, and sequential data analysis. The entire study was conducted in compliance with the Declaration of Helsinki and received approval from the Ethics Committee of West China Hospital.

Clinicopathologic and demographic data, including treatment line, metastatic stage (bone and visceral metastasis), age at sample collection, International Society of Urologic Pathologists (ISUP) grade, and baseline PSA level, were collected. The majority of patients in the hormone‐sensitive prostate cancer (HSPC) stage were treated with maximal androgen blockade (MAB) or MAB plus radiotherapy (34/41, 82.9%), while the remaining four patients received either abiraterone (1/41), docetaxel (1/41), or apalutamide (2/41). In the castration‐resistant prostate cancer (CRPC) cohort, 87.5% (21/24) of patients received novel hormone therapy (abiraterone, enzalutamide, or apalutamide) as their first‐line treatment, with the remaining three patients treated with docetaxel.

The time point of sample collection was also recorded based on the treatment status of patients, specifically the treatment‐naïve, HSPC, and CRPC stages.

### Pathological evaluation

DA is characterized by papillary structures or cribriform glands lined by tall columnar pseudostratified cells arranged in cribriform, papillary, or solid patterns featured by distinctive metastatic behaviors [12]. Patients were diagnosed as having DA based on pathological evaluation of prostatic biopsy samples. The hematoxylin & eosin staining and immunohistochemistry results of prostate cancer with and without DA are presented in supplementary material, Figure S1. The percentage of DA was evaluated by experienced uropathologists according to the fifth edition of the WHO classification of urogenital tumors (supplementary material, Table S1) [12]. For patients receiving both radical prostatectomy (RP) and biopsy, the DA percentage was determined from the RP samples. Notably, patients with other unique morphological features (e.g., intraductal AC of the prostate, neuroendocrine prostate cancer) were excluded from this study.

### Sample processing

Upon receipt, whole blood samples were centrifuged at 1600 *g* for approximately 10 min at room temperature. Following centrifugation, the buffy coat was manually isolated and transferred to new tubes for genomic DNA (gDNA) extraction. Subsequently, the plasma layer was meticulously transferred to 1.5 ml Eppendorf tubes and subjected to a second centrifugation step at 16,000 *g* for another 10 min at 4°C to maximize the elimination of residual cells as well as cell debris.

The isolation of cfDNA from the plasma was carried out using the QIAamp Circulating Nucleic Acid Kit (Qiagen, Hilden, Germany), following the manufacturer's protocol. DNA concentration was quantified using the Qubit dsDNA HS Assay Kit (Thermo Fisher Scientific, Inc., Waltham, MA, USA). DNA extraction from white blood cells was performed using the QIAamp DNA Mini Kit (Qiagen).

### Library generation and sequencing

For the construction of cfDNA libraries, 30–60 ng of cfDNA was utilized with the Kapa Hyper Prep Kit (Kapa Biosystems, Wilmington, MA, USA), incorporating Unique Molecular Identifiers to maximally eliminate background noise. Similarly, gDNA libraries were constructed using the KAPA Hyper Prep Kit (KAPA Biosystems) according to the manufacturer's instructions.

Targeted sequencing of DNA sequences of interest was achieved using a customized set of biotinylated DNA probes and next‐generation sequencing panel kits for both cfDNA and gDNA. The constructed libraries were subsequently loaded onto the Illumina NextSeq 500 platform (Illumina, San Diego, CA, USA) for paired‐end sequencing with 75 base pairs (bp).

### Variant detection procedure, somatic/germline alteration testing, and homologous recombination repair deficiency score estimation

Paired‐end reads obtained from sequencing were mapped using the Burrows‐Wheeler alignment maximal exact matches algorithm. To ensure accurate mutation calling, particularly for variants with low allele frequency in cfDNA, a robust filtering model based on the binomial test was developed. Single‐nucleotide variations (SNVs), small insertions/deletions (Indels), and fusions were called using a heuristic filtering strategy, taking parameters such as base quality, mapping quality, and supporting reads into consideration. Copy number variations in cfDNA were determined using an internally developed method with default parameters. The 90‐gene detection panel with the corresponding pathway is listed in supplementary material, Table S2. Germline alterations were detected using white blood cells, while somatic mutations were detected through cfDNA, respectively. It should be mentioned that for both somatic and germline mutations, only pathogenic or very likely pathogenic variants were selected for further analyses according to American College of Medical Genetics and Genomics criteria [13]. Tumor mutation burden (TMB) scores were calculated based on structural mutations within each megabase pair detected in the whole genome.

To validate the identified mutations, all results underwent manual review utilizing the Integrative Genomics Viewer to eliminate false positives [14]. Additionally, Gaussian kernel smoothing with StatsModels 0.8.0 was employed to accurately calculate the probability density distributions of mutant and wild‐type fragments. Hematopoietic expansion‐related variants were ruled out by comparing to the healthy individual data from the genomic database of West China Hospital by referring to the previous publication [15]. To estimate genomic scars of tumor samples, homologous recombination repair deficiency (HRD) scores were calculated by the unweighted sum of large‐scale state transition (LST), loss of heterozygosity (LOH), and telomeric allelic imbalance (TAI) [16].

### Statistical analysis

Continuous variables were compared using the Wilcoxon–Mann–Whitney test. Fisher's test or chi‐squared test was performed accordingly to assess the genomic differences between DA(+) and DA(−) cohorts. Survival analysis was conducted and plotted as Kaplan–Meier curves. All of the statistical analyses in this study were performed using ‘survival’, ‘survminer’, and ‘ggplot2’ packages, and the heatmap was drawn based on ‘ComplexHeatmap’ and ‘pheatmap’ packages from R (version 4.3.2). The *p* value <0.05 indicated statistical significance.

## Results

### Baseline characteristics of the DA(+) and DA(−) cohorts

**Figure 1:**
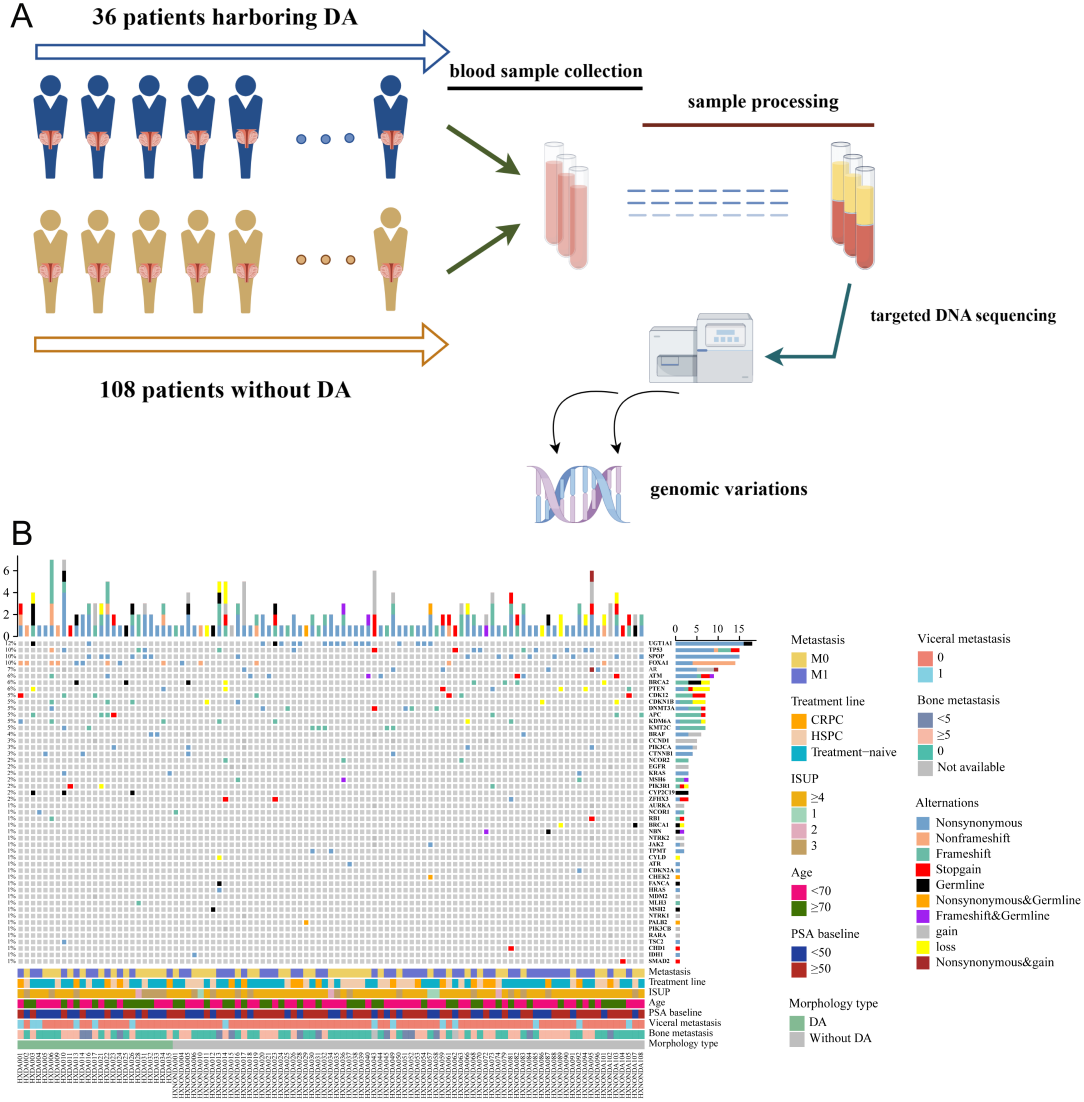
Diagram of the study design and mutational characteristics of the study cohort. (A) Diagram reflecting the general design of the whole study. It is plotted using Figure Draw (version 2.0) and the publication ID is AOWUSdccd4. (B) Heatmap displaying the mutational landscape of the total cohort. CRPC, castration‐resistant prostate cancer; DA, ductal adenocarcinoma of the prostate; HSPC, hormone‐sensitive prostate cancer; ISUP, International Society of Urological Pathology grading; PSA, prostate‐specific antigen; TMB, tumor mutation burden.

A total of 36 patients with DA and 108 patients without DA underwent genomic sequencing (Figure 1A). Baseline characteristics of both cohorts are displayed and compared in Table 1. Generally, patients diagnosed with DA exhibited bone metastatic rates similar to those in the DA(−) cohort at the time of blood sample extraction. However, the DA(+) cohort demonstrated a higher rate of visceral metastasis than the DA(−) cohort (22% versus 7.4%, *p* = 0.028) and significantly lower baseline PSA levels (*p* = 0.007). Other baseline characteristics were well balanced between the two cohorts.

**Table 1:** Baseline characteristics of the DA(+) and DA(−) cohorts

Variable	DA(−), *N* = 108*	DA(+), *N* = 36*	*p* ^†^
Treatment line			0.4
HSPC	34 (31%)	7 (19%)	
CRPC	18 (17%)	6 (17%)	
Treatment‐naive	56 (52%)	23 (64%)	
Metastasis at detection			0.6
M0	52 (48%)	19 (53%)	
M1	56 (52%)	17 (47%)	
Visceral metastasis	8 (7.4%)	8 (22%)	0.028
Bone metastasis			0.7
<5	12 (11%)	6 (17%)	
≥5	38 (35%)	12 (33%)	
0	55 (51%)	18 (50%)	
Not available	3 (2.8%)	0 (0%)	
ISUP			0.055
1	3 (2.8%)	0 (0%)	
2	6 (5.6%)	3 (8.3%)	
3	16 (15%)	11 (31%)	
4	12 (11%)	7 (19%)	
5	71 (66%)	15 (42%)	
Age	68 (61, 73)	68 (61, 73)	>0.9
Baseline PSA			0.007
<50	41 (38%)	23 (64%)	
≥50	67 (62%)	13 (36%)	

CRPC, castration‐resistant prostate cancer; DA, ductal adenocarcinoma of the prostate; HSPC, hormone‐sensitive prostate cancer; ISUP, International Society of Urological Pathology grading; PSA, prostate‐specific antigen.

* *n* (%); median (IQR).

^†^ Pearson's chi‐squared test; Fisher's exact test; Wilcoxon rank‐sum test.

### Mutational features based on liquid biopsy

The detailed somatic and germline mutational profiles of both patients with and without DA are depicted in Figure 1B. Overall, 193 SNVs [59 in DA(+) and 134 in DA(−)] were observed in 101/144 patients. TMB values were also comparable between the DA(+) and DA(−) cohorts (3.2 Mut/Mb versus 4.5 Mut/Mb, *p* = 0.609) (supplementary material, Figure S2A). We performed co‐existing mutation or mutual exclusivity analyses for the total cohort, DA(+) and DA(−) (supplementary material, Figure S2B–S2D), respectively. *UGT1A1* was found to co‐exist with *CYP2C19* in both the DA(−) and the total cohorts. However, in the DA(+) cohort, no top genes (≥5) showed either co‐existence or mutual exclusivity after multitest adjustments (Figure 1B). Pathogenic germline mutation rates were comparable between DA(+) and DA(−) cases (6/36 versus 11/108, *p* = 0.37). In the DA(+) cohort, the most frequent somatic alteration was *FOXA1* (9/36, 25%), followed by speckle‐type POZ protein (*SPOP*) (6/36, 16.7%), CYP2C19 (5/36, 13.9%), and *APC* (4/36, 11.1%). For the DA(−) cohort, the most frequent somatic mutation was *CYP2C19* (19/108, 17.6%), followed by *UGT1A1* (16/108, 14.8%), TP53 (11/108, 10.2%), and *AR* (8/108, 7.4%). Additionally, the *ETS*‐fusion rates between the DA(+) and DA(−) cohorts showed no significant differences (3/36 versus 7/108, *p* = 0.711).

We further investigated the differential genomic alterations between the DA(+) and DA(−) cohorts based on classical PCa‐related pathways, which are discussed in detail in the following paragraphs.

### Comparison of AR pathway mutations

**Figure 2:**
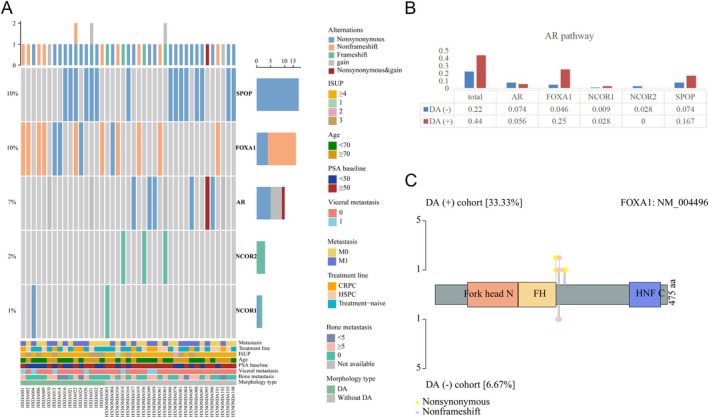
AR pathway mutations of PCa patients with and without DA. (A) Heatmap displaying the AR pathway alterations in tumors with and without DA. (B) Histogram depicting the counts of single‐gene alterations in the AR pathway in the DA cohort. (C) Lollipop plot exhibiting the specific locations of *FOXA1* mutations in our cohort. CRPC, castration‐resistant prostate cancer; DA, ductal adenocarcinoma of the prostate; HSPC, hormone‐sensitive prostate cancer; ISUP, International Society of Urological Pathology grading; PCa, prostate cancer; PSA, prostate‐specific antigen.

The mutational profiles of the AR pathway are displayed in Figure 2A. We found that AR pathway mutations were significantly enriched in DA(+) compared to the DA(−) cohort [16/36 (44.4%) versus 24/108 (22.2%), *p* = 0.017], with 12 (50%) found in the population with DA content ≥10% and 4 (33%) in patients with DA content less than 10%. *FOXA1* [9/36 (25%) versus 5/108 (4.6%), *p* = 0.0012] (Figure 2B,C and supplementary material, Table S3) mutations were enriched in the DA(+) population. Additionally, *NCOR1*, *NCOR2*, and *AR* mutation rates were comparable between the DA(+) and DA(−) cohorts (Figure 2B). In the M1 cohort, the *SPOP* mutation rate was higher in the DA(+) cohort compared with DA(−) [4/17 (23.5%) versus 3/56 (5.4%), *p* = 0.047]. Five out of six *SPOP* alterations were found in patients with DA content ≥10%.

### DNA damage repair pathway

**Figure 3:**
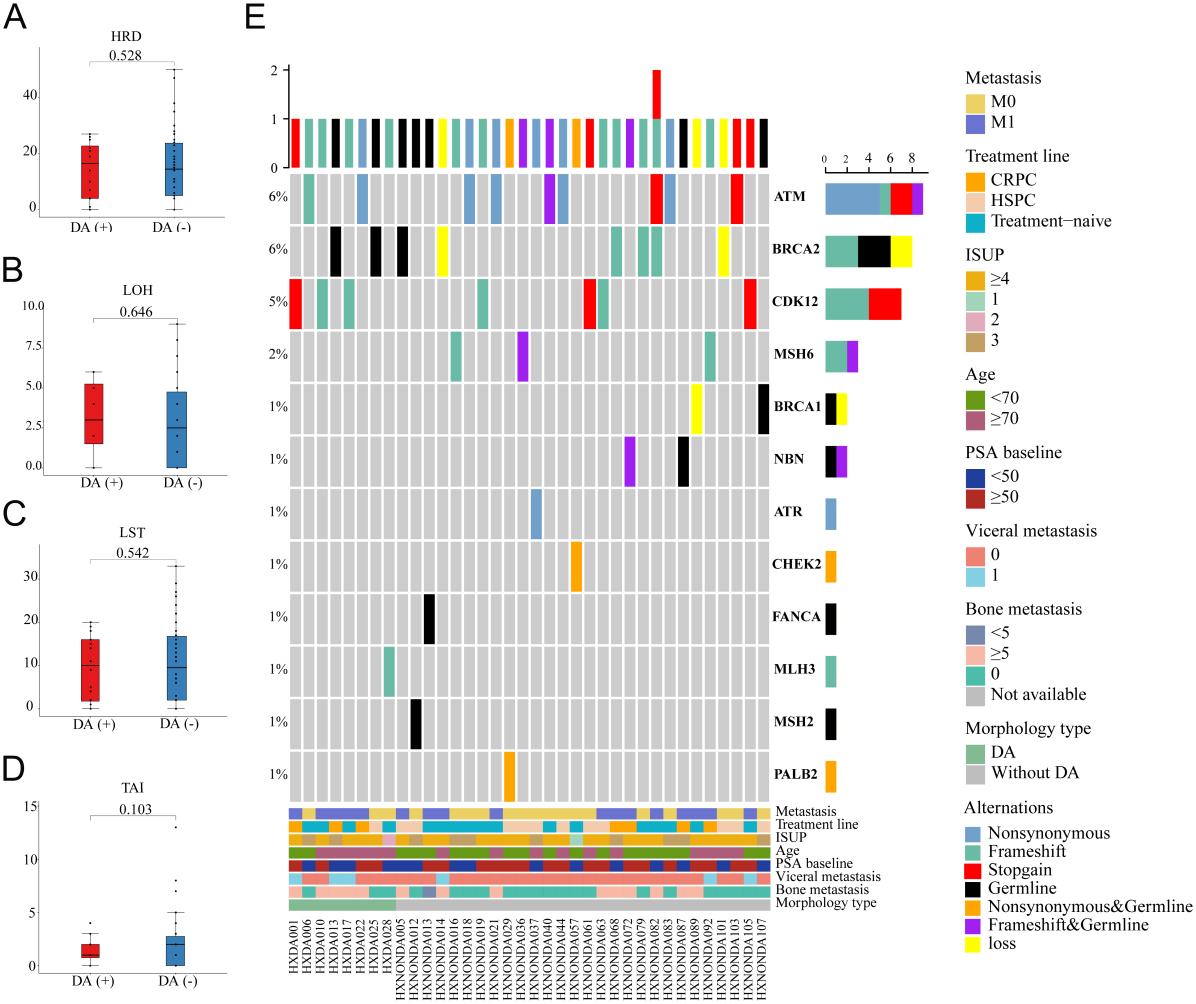
HRD scores and DDR pathway mutations of patients with and without DA. (A) Box plots showing the HRD values of the tumors with and without DA. (B) Box plots showing the LOH values of the tumors with and without DA. (C) Box plots showing the LST values of the tumors with and without DA. (D) Box plots showing the TAI values of the tumors with and without DA. (E) Heatmap displaying the DDR pathway alterations in tumors with and without DA. CRPC, castration‐resistant prostate cancer; DA, ductal adenocarcinoma of the prostate; HRD, homologous recombination repair deficiency; HSPC, hormone‐sensitive prostate cancer; ISUP, International Society of Urological Pathology grading; LOH, loss of heterozygosity; LST, large‐scale state transition; PSA, prostate‐specific antigen; TAI, telomeric allelic imbalance.

The total mutation rate of the DNA damage repair (DDR) pathway was similar between the DA(+) and DA(−) groups [8/36 (22%) versus 27/108 (25%), *p* = 0.825] (Figure 3A). Given that HRD scores more comprehensively reflect the consequence of loss of function in homologous recombination repair than DDR mutations alone [16], we subsequently compared the HRD scores (Figure 3A) between the DA(+) and DA(−) cohorts. The values of LOH (Figure 3B), LST (Figure 3C) and TAI (Figure 3D) were also compared. The results indicated no significant differences between the two cohorts. *BRCA2* germline mutations were also comparable (2/36 versus 1/108, *p* = 0.154).

### WNT pathway

For the WNT signaling pathway, four key genes were targeted for genomic sequencing: *APC*, *CTNNB1*, *RNF43*, and *ZNRF3*. The WNT pathway mutation rate was elevated in the DA(+) cohort compared to the DA(−) cohort [6/36 (16.7%) versus 5/108 (4.6%), *p* = 0.029] (supplementary material, Figure S3A). Five out of six WNT pathway alterations were detected in patients with high DA content (≥10%). The mutation rate of *APC* appeared to be comparable [4/36 (11.1%) versus 3/108 (2.8%), *p* = 0.066] (supplementary material, Figure S3B). Two *CTNNB1* mutations were observed in the DA(+) cohort and the DA(−) cohort [2/36 (5.6%) versus 2/108 (1.9%), *p* = 0.260], respectively (supplementary material, Figure S3A). As for *RNF43* and *ZNRF3*, they were not detected in either the DA(+) or the DA(−) cohort.

### Genome‐deprived prognosticators for the DA(+) and DA(−) cohorts

**Figure 4:**
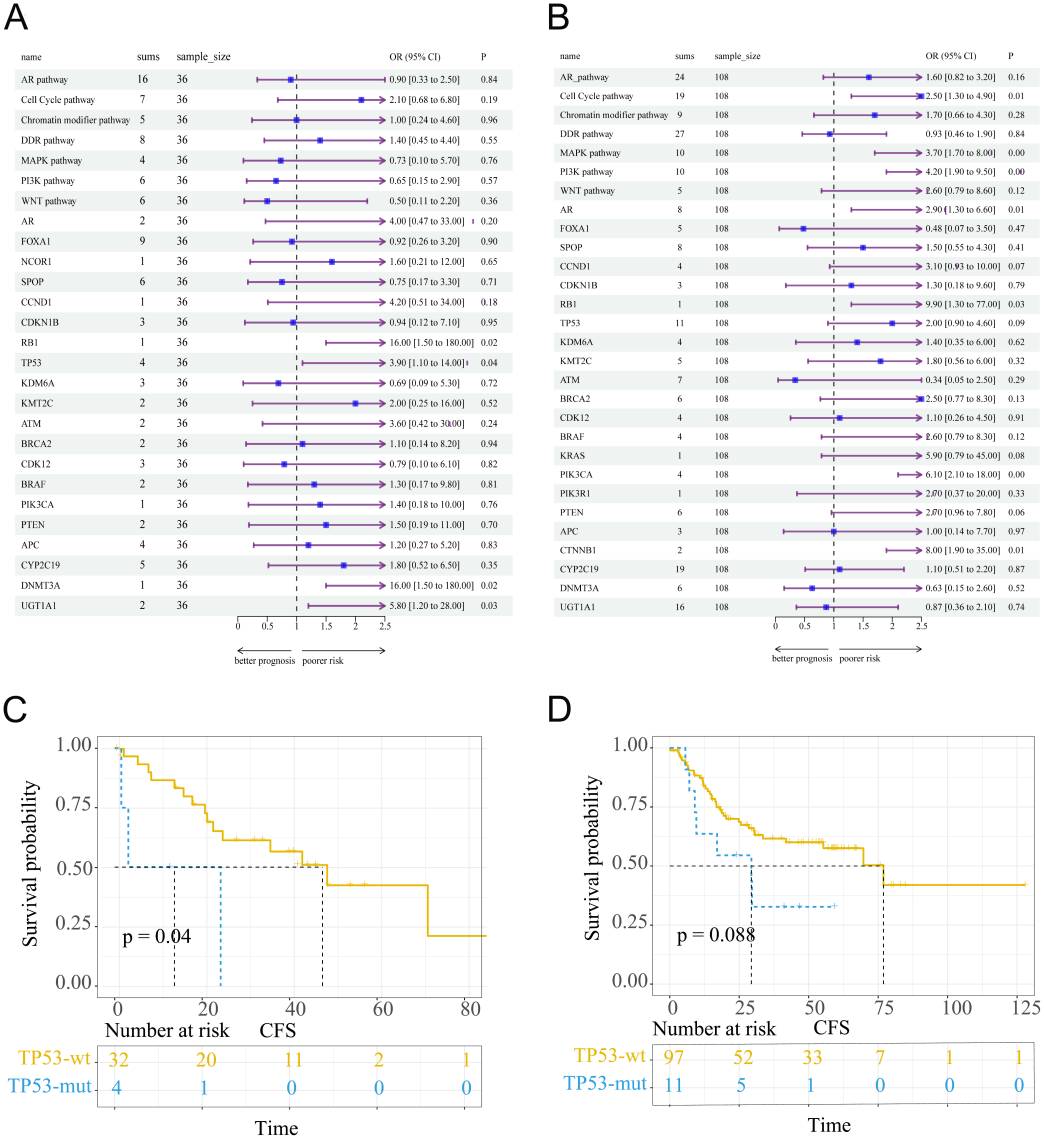
The cfDNA‐derived prognosticators for DA and non‐DA cohorts. (A) Forest plot showing the correlations between genomic mutations and CFS in the DA cohort. (B) Forest plot showing the correlations between genomic mutations and CFS in the non‐DA cohort. (C) Kaplan–Meier curve showing the associations between *TP53* mutational status and CFS in the DA cohort. (D) Kaplan–Meier curve showing the association between *TP53* mutational status and CFS in the non‐DA cohort. CFS, castration‐free survival; DA, ductal adenocarcinoma of the prostate; mut, mutated; wt, wild type.

At the end of follow‐up, 62 (43.1%) patients developed castration resistance. Associations between genomic alterations and castration‐free survival (CFS) in the DA(+) and DA(−) cohorts are displayed in Figure 4A,B, respectively. We found that the *TP53* mutation was associated with shorter CFS (Figure 4C,D) in both the DA(+) (median: 7.9 months versus. 25.7 months, *p* = 0.04) and DA(−) (median: 24 months versus. 28.5 months, *p* = 0.088) cohorts. At the pathway level, cell cycle, MAPK, and PI3K pathway mutations suggested poorer CFS in the DA(−) cohort. However, none of the above correlations were detected in the DA(+) group.

## Discussion

In this study, we conducted a genomic evaluation to assess the mutational profiles of patients with and without DA within the Asian population. AR pathway mutations, particularly *FOXA1*, were significantly enriched in patients diagnosed with DA. WNT pathway mutations were also more commonly observed in the DA(+) cohort. The DDR alteration rates and HRD values were comparable between the two cohorts. Additional findings included the prognostic value of the *TP53* mutation for CFS in both DA(+) and DA(−) cohorts.

Ductal AC is recognized as a rare and aggressive variant of PCa, characterized by unique metastatic patterns and serving as an independent poor prognosticator. The 2022 WHO guidelines debated the reclassification of DA, considering whether DA should be considered a subtype of acinar AC, before maintaining it as a distinct morphological type of PCa rather than integrating it into acinar AC [12]. Additional investigations are warranted regarding the similarities and differences between DA and acinar AC of the prostate.

Serving as the upstream molecular regulator of the AR pathway, *FOXA1* mutations have been reported to potentially induce divergent effects on AR pathway activities—either inhibition or promotion [17]. According to Adams' study, forkhead mutants of F254_E255 and M253_N256 indels in *FOXA1*, which were also observed in our cohort, would induce a pioneering effect by increasing the chromatin opening rate and transcriptional activity, thus potentially facilitating subsequent AR binding [18]. This explained the noninferior or numerically higher AR activity scores observed in DA compared to AC in the Gillard *et al* study [6]. Boysen's team investigated the potential correlations between *SPOP/CHD1* mutational status and abiraterone efficacy. They noted that patients with *SPOP* mutations presented high sensitivity to abiraterone [19]. In our study, we have found that, in the M1 cohort, the DA(+) group harbored a significantly higher *SPOP* mutation rate than the DA(−) group. Though highly hypothesis‐generating, we assume that a higher *SPOP* mutation rate in metastatic DA(+) might contribute to its considerable response to abiraterone [7].

Enrichment in WNT‐pathway mutations is associated with PCa progression, metastasis, and castration resistance [20]. It is also relevant to the cribriform morphogenesis of PCa [6]. Sutera *et al* indicated that the WNT pathway was strongly associated with a higher visceral metastasis rate [21], with *APC* mutation being especially notable in the Chinese population [22]. We observed that the WNT pathway mutations, mainly *APC*, were more frequent in the DA(+) tumors compared to the DA(−) PCa, thereby partially explaining the higher visceral metastasis rate observed in the DA(+) population from a genomic perspective. Similar findings reported in other studies further corroborate our results [6, 8].

Unlike some studies that linked DA with an increased detection rate of DDR mutation [9], we observed no significant differences in DDR pathway mutations between patients with or without DA regardless of their metastatic stage. This finding aligns with Kobayashi *et al*'s study, which reported no DDR alterations in any samples from aggressive DA [8]. While HRD scores suggest genomic scars and reflect the consequences of loss of function in homologous recombination repair in tumor samples [16], the HRD scores were also comparable between the DA(+) and DA(−) groups. Consequently, we hypothesized that PCa with ductal morphology may not feature a frequent occurrence of DNA damage repair deficiency.

This study should be interpreted with several limitations. First, the sample size in the DA(+) cohort was relatively small due to the rarity of PCa with ductal morphology, restricting the robustness of our findings. Second, samples collected from DA(+) and DA(−) were based on a 12‐core prostate biopsy, and some cases presenting coexistence of both DA and prostate AC may cause bias.

In conclusion, DA was characterized by a higher AR pathway mutation rate compared to DA(−) PCa, typically involving the *FOXA1* mutation. Additionally, the DA(+) cohort harbored more WNT pathway mutations. The DDR mutation rate and HRD scores were comparable between the DA(+) and DA(−). *TP53* mutation served as a deteriorating prognosticator for patients with and without DA in terms of CFS. Further large‐scale studies are warranted for additional validation.

## Author contributions statement

QZ, TW, YS and XZ provide equal contributions to this study. Conception and design: QZ, JZ; acquisition of data: QZ; analysis and interpretation of data: QZ; drafting of the manuscript: QZ, YS, TW, DL, CX; critical revision of the manuscript for important intellectual content: JC, HL, FZ, YC, JL, NC, PS, HZ, JZ; statistical analysis: QZ; obtaining funding: HZ; administrative, technical or material support: HZ; supervision: HZ.

## Supporting information


**Figure S1.** HE staining and immunohistochemistry of prostate cancer with and without ductal morphology
**Figure S2.** TMB and mutational profiles
**Figure S3.** WNT pathway mutations

**Table S1.** DA percentage for each patient receiving liquid biopsy

**Table S2.** cfDNA sequencing panel

**Table S3.** Comparison of mutation profiles between patients with and without DA

## Data Availability

Related files for generation of the results are available upon reasonable request.
