# Supplementary material for: Genomic features of liquid biopsies from patients with prostate cancer with and without ductal adenocarcinoma
Source: J Pathol Clin Res. 2025 Apr 2;11(3):e70026. doi: 10.1002/2056-4538.70026 (PMC11963801; doi:10.1002/2056-4538.70026)
Supplement: Supplementary file 1 — Figure S1. HE staining and immunohistochemistry of prostate cancer with and without ductal morphology Figure S2. TMB and mutational profiles Figure S3. WNT pathway mutations [file CJP2-11-e70026-s003.pdf]

Genomic features of liquid biopsies from patients with prostate cancer with and without ductal adenocarcinoma

Q Zhu, T Wang, Y Shi, X Zhou *et al. J Pathol Clin Res*
https://doi.org/10.1002/2056-4538.70026

Supplementary Figures S1–S3

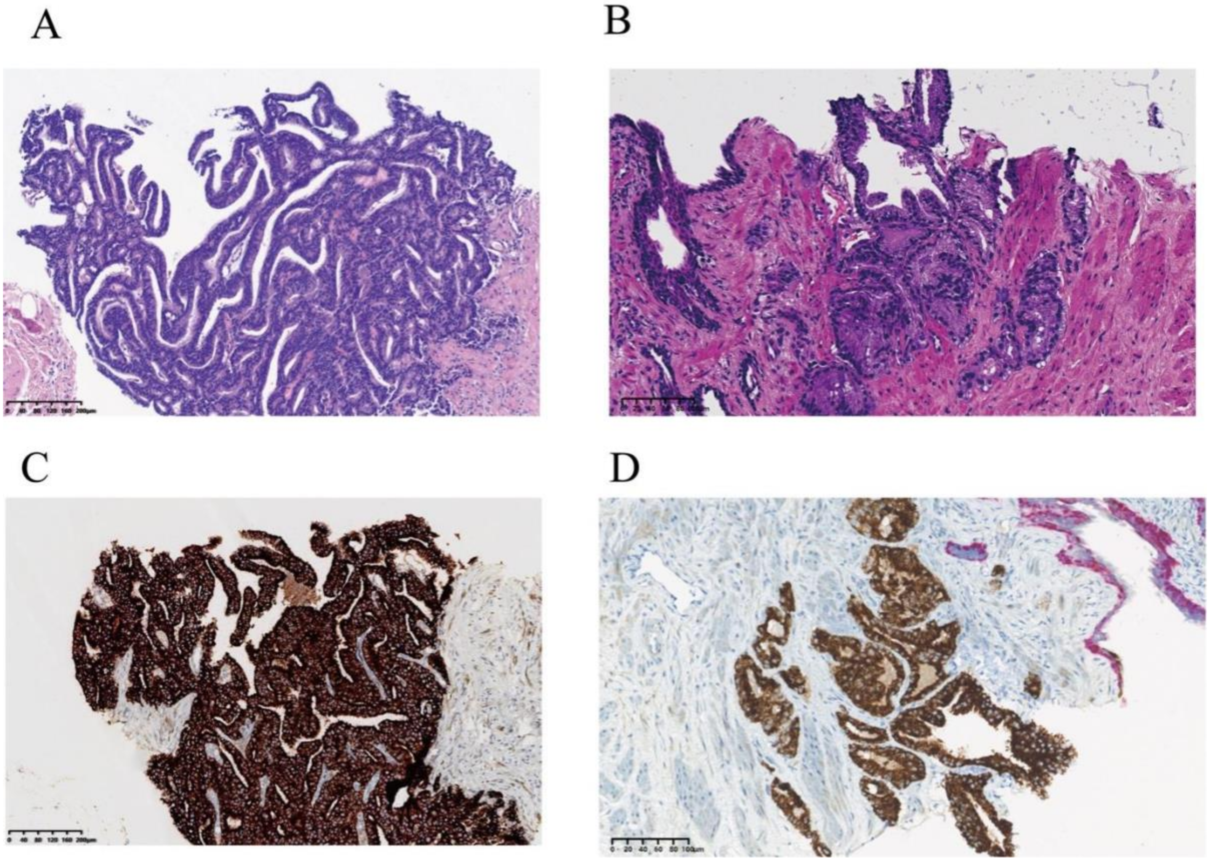

Figure S1. HE staining and IHC of prostate cancer with and without ductal morphology. (A) HE staining of ductal adenocarcinoma of the prostate. (B) HE staining of non-ductal adenocarcinoma of the prostate. (C) IHC staining for AMACR/HCK/p63 in ductal adenocarcinoma of the prostate. (D) IHC staining for AMACR/HCK/p63 in non-ductal adenocarcinoma of the prostate. In (C) and (D), AMACR is positive (brown) in the cytoplasm of prostate cancer cells, while HCK and p63 are positive (red) in the cytoplasm and nucleus respectively of basal cells. Abbreviations: HE: Hematoxylin and Eosin Staining; IHC: immunohistochemistry; AMACR: alpha-methylacyl-CoA-racemase; HCK: high molecular weight cytokeratin.

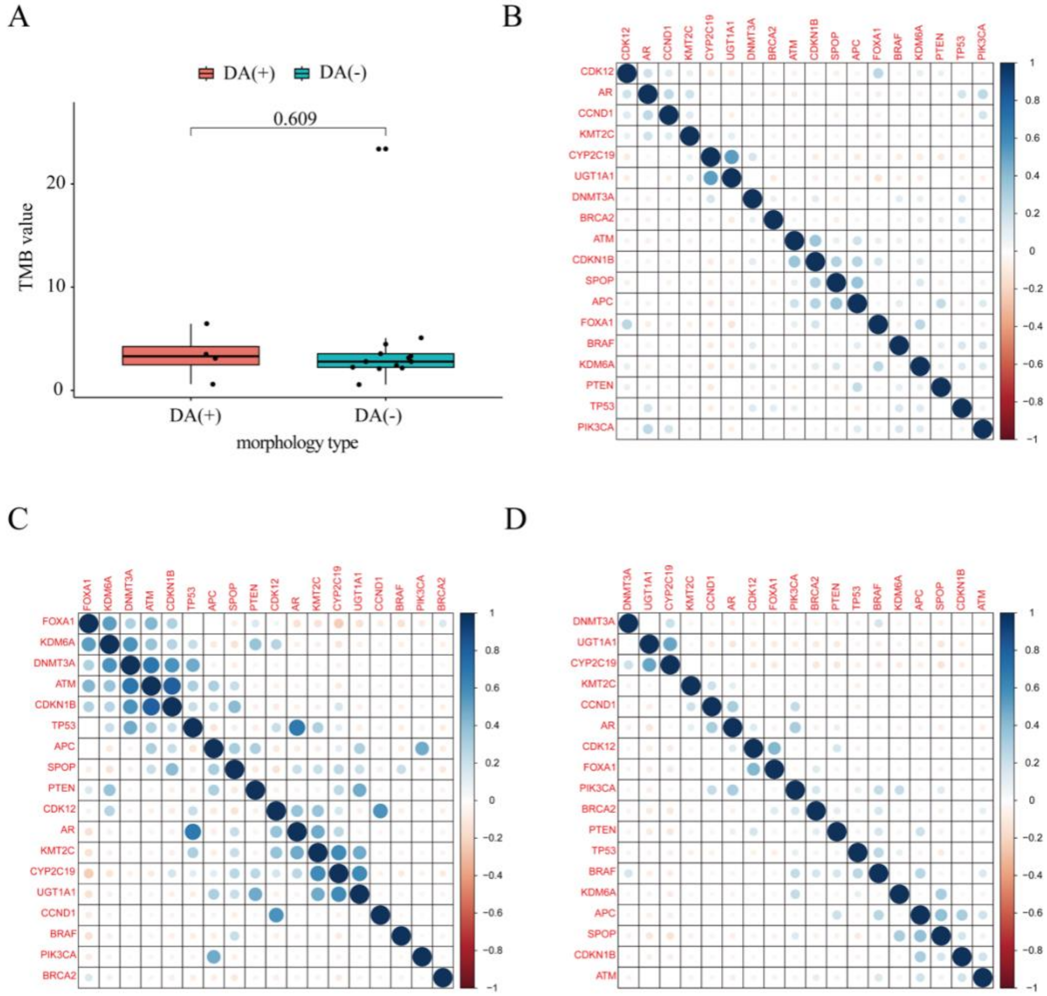

Figure S2. TMB and mutational profiles. (A) Box plots showing the TMB values of the tumors with and without DA. (B) Coexistence and mutually exclusive analysis of mutational profiles of the total cohort. (C) Coexistence and mutually exclusive analysis of mutational profiles of the DA cohort. (D) Coexistence and mutually exclusive analysis of mutational profiles of the non-DA cohort. Abbreviations: TMB: tumor mutation burden; DA: ductal adenocarcinoma.

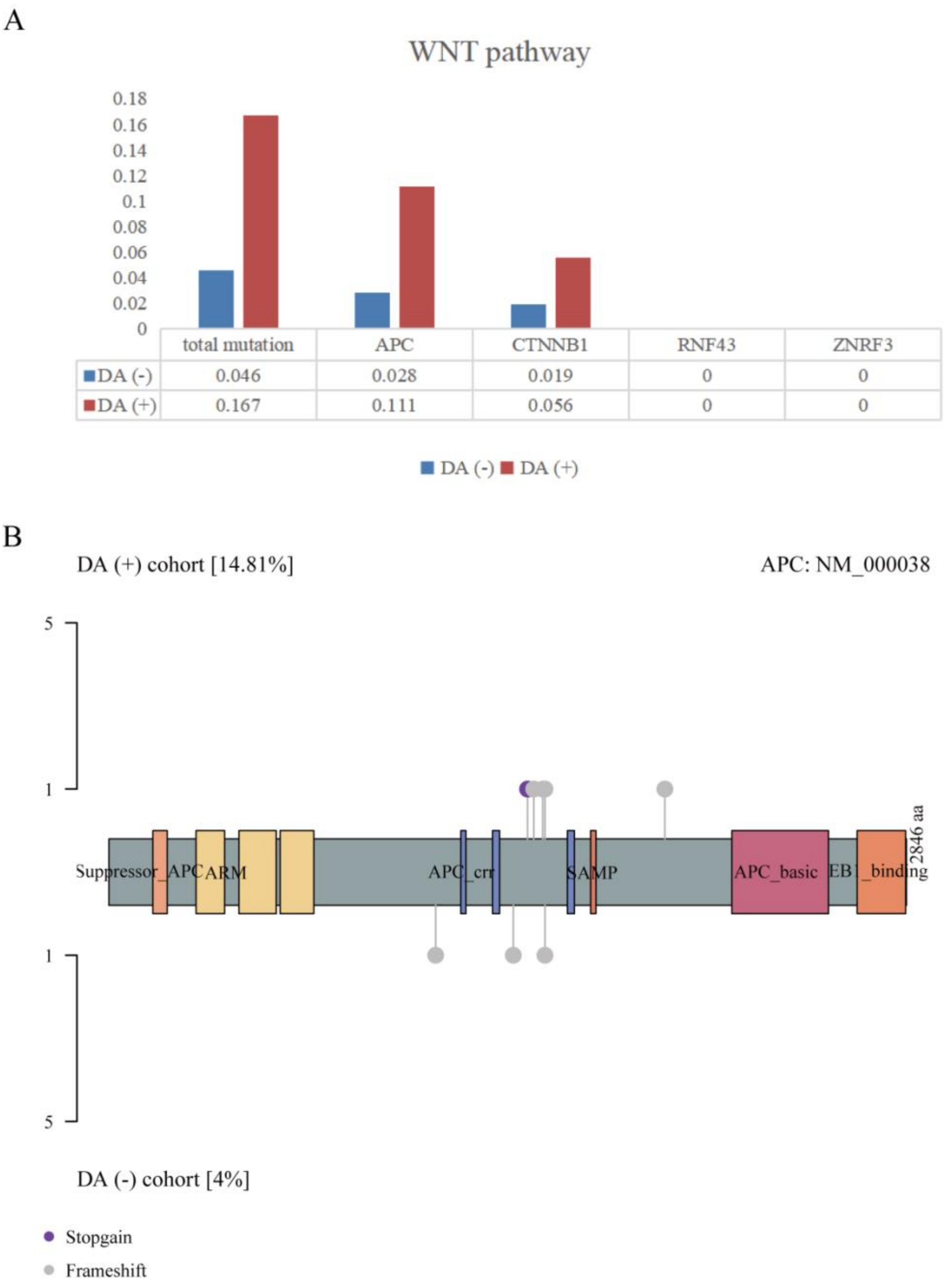

Figure S3. WNT pathway mutations. (A) Histogram depicting the counts of single gene alterations in WNT pathway in the DA cohort. (B) Lollipop plot exhibiting the specific locations of *APC* mutations in our cohort.
